# Concurrent presentation of proliferative glomerulonephritis with monoclonal immunoglobulin deposits and light chain proximal tubulopathy: a case report and review of the literature

**DOI:** 10.3389/fmed.2025.1502798

**Published:** 2025-01-31

**Authors:** Jingdong Zhang, Yang Liu, Fengyan Jin, Jia Li, Hui Wang, Fuzhe Ma, Ye Jia, Jinyu Yu, Shan Wu, Shaojie Fu, Zhonggao Xu, Hao Wu

**Affiliations:** ^1^Department of Nephrology, The First Hospital of Jilin University, Changchun, China; ^2^Department of Hematology, The First Hospital of Jilin University, Changchun, China; ^3^Laboratory of Electron Microscopy, Peking University First Hospital, Beijing, China

**Keywords:** proliferative glomerulonephritis with monoclonal immunoglobulin deposits, light chain proximal tubulopathy, monoclonal gammopathy, nephrotic syndrome, renal biopsy, case report

## Abstract

The simultaneous occurrence of proliferative glomerulonephritis with monoclonal immunoglobulin deposits (PGNMID) and light chain proximal tubulopathy (LCPT) presents a unique diagnostic and therapeutic challenge. PGNMID is characterized by monoclonal immunoglobulin deposition in glomeruli, leading to proliferative glomerular pathology, while LCPT involves monoclonal light chain deposition in proximal tubular cells, causing tubulointerstitial damage. Both conditions are classified under monoclonal gammopathy of renal significance (MGRS), but their coexistence in a single patient is exceedingly rare. This case report details the presentation of a patient with nephrotic syndrome and renal insufficiency, where renal biopsy revealed both PGNMID and LCPT. Treatment with bortezomib, cyclophosphamide, and dexamethasone achieved clinical remission and significant renal function recovery. This case emphasizes the critical role of renal biopsy in the diagnosis, particularly in the absence of detectable monoclonal proteins, and demonstrates the efficacy of targeted therapy in managing such complex renal pathologies. These findings contribute to a better understanding of MGRS and may guide future therapeutic strategies for similar cases.

## Introduction

MGRS encompasses disorders that cause kidney damage due to monoclonal gammopathy, even when no hematologic malignancies are evident. Among the various manifestations of MGRS, PGNMID, and LCPT stand out as distinct and complex subtypes. PGNMID, a proliferative glomerulonephritis, occurs in 0.17 to 3.7% of renal biopsies, characterized by granular monoclonal immunoglobulin deposits (1). It presents significant clinical challenges due to its rarity and aggressive progression, with approximately 38% of patients developing chronic kidney disease (CKD) and 22% progressing to end-stage renal disease (ESRD) (2). LCPT, which appears in only approximately 0.06% of renal biopsies (3), is marked by the accumulation of microcrystalline or amorphous inclusions within the proximal renal tubular cells, further complicating the clinical scenario (4). The concurrent presence of PGNMID and LCPT is rare, posing unique diagnostic and therapeutic challenges. This report describes a case of a 71-year-old Asian male with these concurrent conditions, treated with bortezomib, cyclophosphamide, and dexamethasone, achieving significant remission after five treatment cycles. This case underscores the need for accurate diagnosis and targeted treatment to address the underlying pathogenic drivers in MGRS and optimize patient outcomes.

## Case report

A 71-year-old Asian male sought medical treatment due to bilateral lower limb edema. Initial examination revealed microscopic hematuria and significant proteinuria, with a 24-h urinary protein excretion of 6.492 g. Laboratory results indicated a serum albumin level of 24.5 g/L, serum creatinine of 138.7 μmol/L, and an estimated glomerular filtration rate (eGFR) of 43.73 mL/min/1.73m^2^. Complement C3 level was low at 0.61 g/L. Comprehensive serological tests for autoimmune diseases, infectious diseases, and malignancies, including ANA, anti-dsDNA, anti-ENA, anti-GBM, ANCA, hepatitis B and C, syphilis, HIV, and cancer-specific tumor markers, returned negative. Serum and urine immunofixation electrophoresis did not detect monoclonal proteins.

Table 1 provides the recorded values for immunoglobulin light chains, with urine kappa light chains measured at 192.00 mg/24 h (standard range: 0–14.2 mg/24 h), and urine lambda light chains at 88.05 mg/24 h (standard range: 0–7.8 mg/24 h). In the serum, free kappa chains were found to be 42.80 mg/L (standard range: 6.7–22.4 mg/L), and free lambda chains were 43.80 mg/L (standard range: 8.3–27.00 mg/L). The urinary kappa/lambda ratio of 0.79, within normal limits, suggests that these light chain elevations are not indicative of primary tubular proteinuria. The predominant presence of albumin in the urine further supports a diagnosis of primarily glomerular proteinuria, rather than tubular dysfunction.

**Table 1:** Clinical data of the patient at the time of hospital admission.

Parameter	Value	Normal range
**Complete blood count**		
White blood cell count (×10^9^ /L)	5.45	3.5–9.5
Red blood cell count (×10^12^ /L)	3.41	4.3–5.8
Hemoglobin (g/L)	107	130–175
Platelets (×10^9^ /L)	248	125–350
**Biochemistry**		
Total protein (g/L)	42	65–85
Albumin (g/L)	24.5	40–55
Urea nitrogen (mmol/L)	7.34	3.6–9.5
Creatinine (μmol/L)	139.7	53–115
Uric acid (μmol/L)	490	210–430
Calcium (mmol/L)	2.06	2.2–2.65
Inorganic phosphate (mmol/L)	1.17	0.85–1.51
Total cholesterol (mmmol/L)	6.86	2.6–6.0
Triglycerides (mmmol/L)	1.26	0.28–1.80
LDL-C (mmol/L)	4.42	2.06–3.10
**Immunological test**		
C3 component (g/L)	0.61	0.7–1.4
C4 component (g/L)	0.13	0.1–0.4
ANA	Negative	Negative
ds-DNA	Negative	Negative
ANCA	Negative	Negative
SPEP	No M spike	No M spike
SIFE/UIFE	Negative	Negative
Free κ/λ ratio	0.79	0.26–1.65
Cold agglutinin test	Negative	Negative
**Infection markers**		
HBsAg	Negative	Negative
HCV antibody	Negative	Negative
HIV	Negative	Negative
TPA	Negative	Negative
**Urinalysis**		
Red blood cell count (/HPF)	23	0–5
24 h urine protein (g)	6.482	<0.15
24 h urine κ (mg)	88.05	0–7.8
24 h urine λ (mg)	192	0–14.2
24 h urine β2-MG (mg)	0.17	0–0.4
24 h urine phosphate (mmol)	17.5	22–48

ANA, antinuclear antibody; ANCA, anti-neutrophil cytoplasmic antibodies; anti-dsDNA, anti-double stranded DNA; eGFR, estimated glomerular filtration rate; HBsAg, hepatitis B surface antigen; HCV, hepatitis C virus; HIV, human immunodeficiency virus; HPF, high-power field; LDL-C, low-density lipoprotein cholesterol; SIFE, serum immunofixation electrophoresis; SPEP, serum protein electrophoresis; TPA, treponema pallidum antibody; UIFE, urine immunofixation electrophoresis.

**Figure 1:**
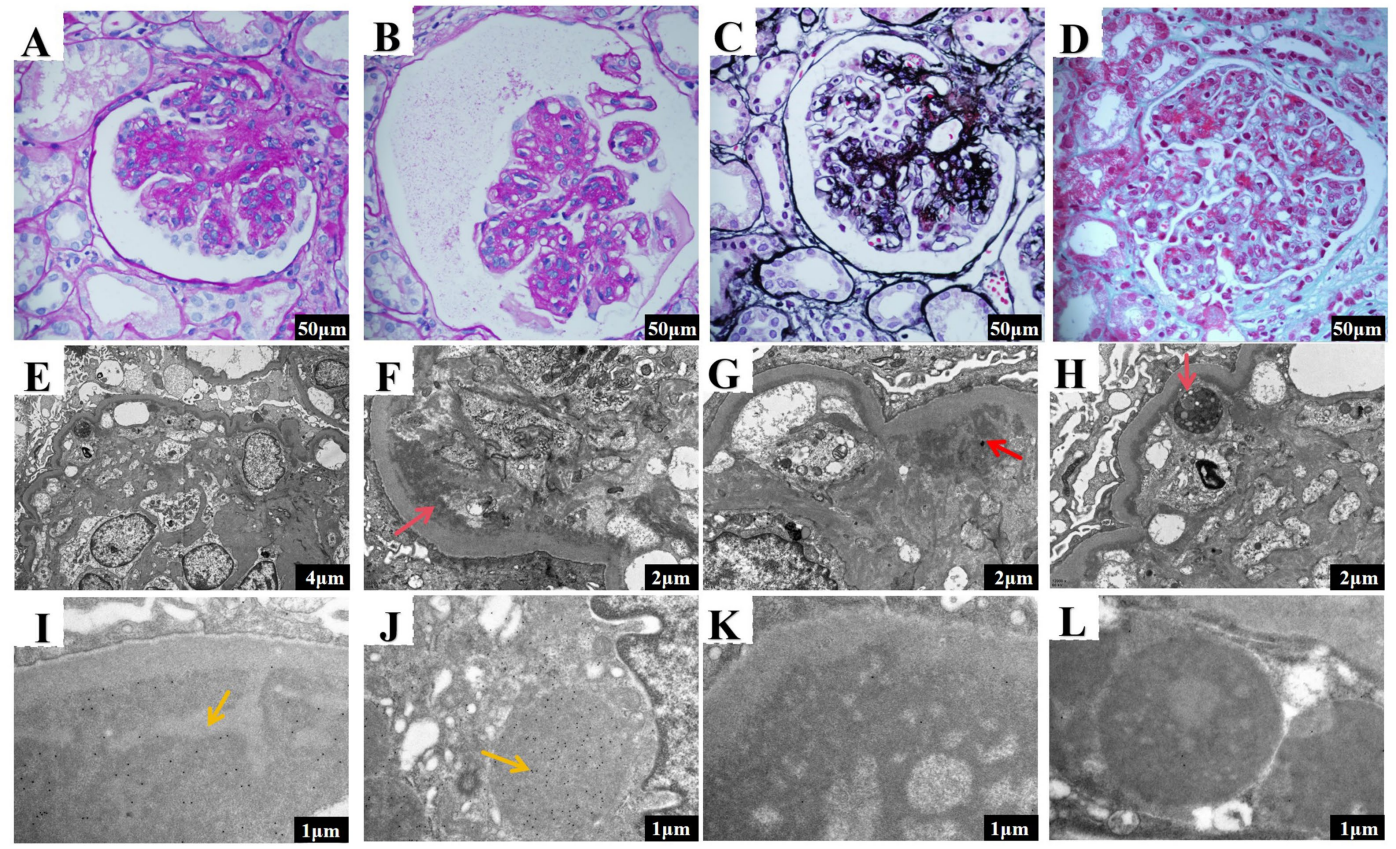
Renal pathology results of the patient in the case report. **(A,B)** The glomeruli exhibit mesangial and endocapillary hypercellularity (scale bar 50 μm). **(C)** Eosinophilic material deposits are visible in the subepithelial, subendothelial, and mesangial areas (scale bar 50 μm). **(D)** The basement membrane is thickened, showing a diffuse double-contour structure (scale bar 50 μm). **(E)** Moderate to severe proliferation and extensive lobulation in mesangial cells and matrix, along with a segmental increase in intracapillary cells (scale bar 4 μm). **(F)** Transmission electron microscopy revealed moderate to severe mesangial cell and matrix hypercellularity, with red arrows indicating subendothelial electron-dense deposits and widespread interposition (scale bar 2 μm). **(G)** Transmission electron microscopy showed segmental capillary lumen cellularity, with red arrows indicating mesangial electron-dense deposits, significant basement membrane thickening, and double contours (scale bar 2 μm). **(H)** Increased lysosomes were observed in proximal tubular epithelial cells, with red arrows pointing to the areas of lysosome accumulation (scale bar 2 μm). **(I)** Ig Kappa staining revealed electron-dense deposits in the mesangial and subendothelial regions of the glomeruli (scale bar 1 μm). **(J)** Ig Kappa staining revealed electron-dense deposits in the proximal tubular epithelial cells (scale bar 1 μm). **(K)** Ig Lambda staining reveals the absence of electron-dense deposits in the glomeruli (scale bar: 1 μm). **(L)** Ig Lambda staining also indicates no electron-dense deposits in the proximal tubular epithelial cells (scale bar: 1 μm).

A renal biopsy revealed that 8 of 25 glomeruli had undergone sclerotic changes, while the remaining evidenced significant mesangial cell proliferation and matrix expansion, extensive lobulation, and double contours of the basement membranes. HE staining indicated mesangial and endothelial cell proliferation (Figures 1A,B). Masson’s trichrome staining highlighted eosinophilic deposits in various glomerular regions (Figure 1C), which tested negative for Congo red staining, indicating non-amyloid material. PASM staining demonstrated basement membrane thickening and diffuse double contours (Figure 1D). Additionally, the tubular epithelial cells demonstrated focal granular and vacuolar degeneration, brush border detachment, and focal atrophy. Immunofluorescence revealed granular deposits of IgG, C3, C1q, IgG3, kappa light chains within the glomeruli. Electron microscopy confirmed membranoproliferative glomerulonephritis (MPGN) and revealed an accumulation of lysosomes in tubular epithelial cells (Figures 1E–H) while immunoelectron microscopy identified kappa electron-dense deposits predominantly in subendothelial and mesangial areas of the glomeruli as well as within tubular cell lysosomes (Figures 1I,J). Lambda deposits were not observed (Figures 1K,L). The final pathological diagnosis identified PGNMID (IgG3*κ*) coexisting with LCPT (κ). These results suggest that the proteinuria primarily originates from glomerular dysfunction. Supporting this, electron microscopy identified kappa light chain deposits within proximal tubular epithelial cells, along with lysosomal accumulation and localized epithelial degeneration. These findings are indicative of secondary tubular injury resulting from the substantial burden of light chains in the glomeruli.

Bone marrow biopsies were conducted under sterile conditions from both the iliac crest and sternum, and analyzed by two independent hematopathologists with expertise in bone marrow pathology. In instances of differing opinions, additional smears were prepared and jointly reviewed to reach a consensus. Histological evaluation involved hematoxylin and eosin staining to examine marrow architecture and cellularity, while immunohistochemical staining with CD138 was employed to identify plasma cells and evaluate monoclonality. Flow cytometry analysis was performed on bone marrow aspirates, using markers for B cells (CD19, CD20), plasma cells (CD138, CD38), and T cells (CD3, CD4, CD8). To ensure precision, gating strategies, fluorescence-minus-one controls, and a normal donor bone marrow sample as a negative control were utilized.

The integrated results from histology, immunohistochemistry, and flow cytometry indicated active marrow proliferation but did not reveal any morphological abnormalities, or monoclonal plasma cells, thereby excluding hematological proliferative disorders. CT imaging corroborated these findings, showing no lymphadenopathy, masses, or other abnormalities indicative of lymphoma. Peripheral blood flow cytometry confirmed the absence of clonal B cells. Although a PET-CT scan might have provided further validation, it was foregone due to patient refusal.

Further diagnostic assessments confidently excluded systemic lymphoproliferative disorders, including indolent lymphoma. Other potential tubular diseases were systematically ruled out. Myeloma cast nephropathy and related multiple myeloma tubular diseases were dismissed due to the lack of clonal plasma cells in the bone marrow biopsy and negative serum/urine immunofixation electrophoresis. The low urinary phosphate level (17.5 mmol/24 h) effectively eliminated Fanconi syndrome, supported by the absence of other signs of proximal tubular dysfunction such as glucosuria or decreased urine specific gravity. Additionally, drug-induced nephrotoxicity was ruled out, as there was no exposure to nephrotoxic drugs, and genetic tubular disorders like Dent disease or Gitelman syndrome were considered unlikely due to the lack of characteristic metabolic abnormalities, such as hypercalciuria and hypokalemia.

**Figure 2:**
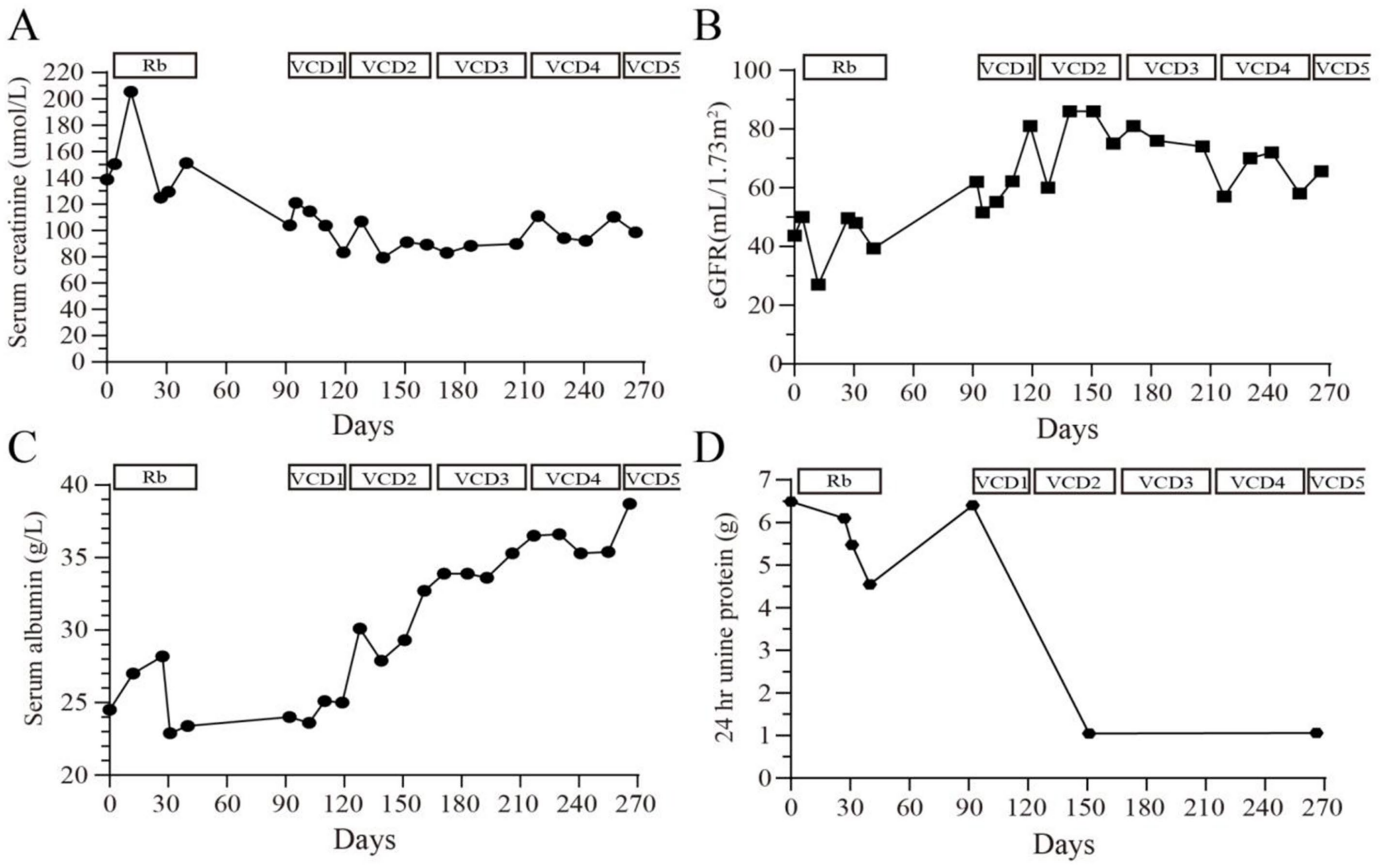
Changes in **(A)** serum creatinine (μmol/L), **(B)** estimated glomerular filtration rate (mL/min/1.73m^2^), **(C)** serum albumin (g/L) and **(D)** 24-h urinary protein quantification (g) during the 270 days of treatment and follow-up. Rb, rituximab; VCD, bortezomib, cyclophosphamide, dexamethasone.

To address the MGRS, a chemotherapy regimen known as VCD—comprising bortezomib (V), cyclophosphamide (C), and dexamethasone (D)—was implemented over five cycles. The regimen was administered as follows: bortezomib: 1.3 mg/m^2^ on day 1, 8, 15, and 22; cyclophosphamide: 300 mg/m^2^ on day 1, 8, and 15; dexamethasone: 20 mg on day 1–2, 8–9, 15–16, and 22–23. Throughout nearly 300 days of monitoring, significant improvements were noted post-treatment. By day 270, urinary protein levels had decreased dramatically from 6.492 g/24 h to 1.056 g/24 h, and serum creatinine levels fell from 205.5 μmol/L to 98.5 μmol/L (Figures 2A–D). Urinary β2-microglobulin levels were utilized as markers to assess renal tubular damage. Initially, the levels were recorded at 0.7 mg/24 h. Over time, these levels increased to 1.12 mg/24 h, indicative of tubular injury. After initiating treatment, there was a decrease in these levels to 0.67 mg/24 h, indicating an improvement in tubular function and suggesting a positive response to the treatment regimen.

## Discussion

PGNMID and LCPT, both subtypes of MGRS, display characteristics common to both hematological and renal disorders. PGNMID can affect any age groups but predominantly appears in individuals over the age of 50, with over half of these cases advancing to CKD or ESRD. Pathologically, PGNMID is most often characterized by MPGN. However, it has also been reported to present as intracapillary proliferative glomerulonephritis, crescentic glomerulonephritis, membranous nephropathy, and mesangial proliferative glomerulonephritis as well (5, 6).

The exact cause of PGNMID remains unclear, yet it is theorized to involve the accumulation of IgG proteins, particularly the IgG3 subtype, within the glomeruli plays a significant role (7, 8). Although immunoglobulin deposits are commonly observed in renal pathology associated with PGNMID, many cases show no detectable signs of monoclonal gammopathy or hematologic malignancy, indicating potential shortcomings in current diagnostic methods or that immunoglobulins might only be deposited in the kidney (9). Additionally, PGNMID frequently reappears in allografts soon after transplantation (10), hinting at a process driven by an intrinsic clonal expansion of B cells or plasma cells that excessively produce monoclonal immunoglobulins. This excessive production can quickly accumulate in the glomeruli, leading to further inflammation and damage.

Diagnosing PGNMID presents notable challenges, primarily due to the absence of detectable immune complexes or monoclonal proteins in standard blood and urine screenings. Lin and Chen (11) indicated that only 25% of PGNMID patients demonstrated clonal abnormalities in bone marrow, with 50% showing plasma cell clones detected through immunohistochemistry and flow cytometry, and the others showing CD20+ clones. For these patients, employing more sensitive or specific methods such as immunohistochemistry, flow cytometry, and cytogenetics may be crucial for identifying potentially inactive or dispersed clones.

Bortezomib, essential for treating MGRS-related diseases, inhibits the 26S proteasome to induce apoptosis in cells with misfolded proteins and exhibiting anti-fibrotic properties that benefit kidney health (12). While specific data on bortezomib’s effectiveness in PGNMID are sparse, some reports highlight its successful application in clinical settings. The International Kidney Myeloma Group (IKMG) (13) suggests tailoring PGNMID treatment to disease severity, recommending symptomatic treatment for early-stage CKD with minimal urinary protein and no progression, while advising a regimen of cyclophosphamide and bortezomib for more advanced stages or higher protein levels. Lin and Chen (11) reported a 100% remission rate in patients with identifiable clones who received monoclonal chemotherapy, while a 90% remission rate was observed in those treated empirically without detectable clones. Research by Gumber et al. (14) found that 37% of patients undergoing immunomodulatory therapy had detectable circulating immunoglobulins, 32% had detectable clonal plasma cells or B cells, with 81% responding well to the therapy, and 88% achieving renal remission (Table 2).

**Table 2:** Proliferative glomerulonephritis with monoclonal immunoglobulin deposits: bortezomib-based treatment regimens and responses.

Author, reference	Age/sex	(A)SCr (umol/L)	(A)UP(g/24 h) or (g/gCr*)	Light microscopy	Deposited immunoglobulin	Treatment	(B)SCr(umol/L)	(B)UP(g/24 h) or (g/gCr*)	Renal response	Detectable clone on bone marrow biopsy	Serum/urine M protein
Noto, ‍R‍ et al. (2017)	75/M	134.3	6	MPGN	IgG3-κ	VD	88.4	0.25*	CR	Yes	IgGκ
Gumber, R‍ et al. (2018)	53/M	150.3	3	MPGN	IgG-*κ*	VCD	NA	NA	CR	Plasma cell (5–10%)	IgGκ
Gumber, R‍ et al. (2018)	69/M	159.1	3.43	MPGN	IgG-ʎ	VCD + RTX	NA	NA	CR	No	IgGʎ
Gumber, R et al. (2018)	75/F	274	1.47	MGPN and ECPGN	IgG-κ	VD	NA	NA	PR	No	No
Yu, X‍ et al. (2019)	50/F	264.3	12.3	MPGN	IgG3-ʎ	VCD + RD	132.6	0.8	PR	No	No
Lee,‍ H‍ et al. (2019)	61/F	79	2.01	MPGN	IgG4-κ	VCD	60–80	0.18–0.29	CR	No	No
Quattrocchio, G‍ et al. (2020)	59/M	132.6	2.7	MPGN	IgG3-κ	VCD	NA	0.05	CR	No	No
Quattrocchio, G‍ et al. (2020)	71/F	NA	7	MPGN	IgG3-ʎ	VCD	NA	0.23	CR	No	No
Xu,‍ Z‍ et‍.al. (2021)	27/F	120	3.09	MPGN	IgG3-κ	VCD	111	1.4*	PR	No	No
Rahbari, E‍ et al. (2022)	66/M	159.1	4.3	MPGN	IgA-κ	VCD	2.3	3.5	PRD	No	IgAκ
Present‍ case	71/M	138.7	6.492	MPGN	IgG3-κ	VCD	98.5	1.056	PR	No	No

C, cyclophosphamide; D, dexamethasone; ECPGN, endocapillary proliferative glomerulonephritis; F, female; M, male; MPGN, membranoproliferative glomerulonephritis; NA, not available; R, lenalidomide; RTX, rituximab; SCr, serum creatinine; V, bortezomib; A, before treatment; B, after treatment; CR, complete remission, complete remission is defined as the remission of proteinuria to <0.5 g/day with normal renal function; PR, partial remission, partial remission is defined as >50% decrease but >0.5 g/day in proteinuria with stable eGFR; PRD, did not meet CR or PR criteria, but did not reach ESRD; ESRD, end stage renal disease.

In this case, renal pathology confirmed the presence of a monoclonal light chain-related renal disease known as non-crystalline LCPT. LCPT is marked by the deposition of monoclonal light chains within the proximal renal tubules, forming either crystalline or non-crystalline inclusions (4). IKMG (13) treatment recommendations for LCPT emphasize reducing monoclonal light chain production using a strategy similar to multiple myeloma treatment, including bortezomib and other chemotherapeutics. This approach has shown effectiveness in clinical practice (15), with research such as the study by Wang et al. (16) demonstrating notable improvements in proximal tubular function in about 62.5% of LCPT patients treated with chemotherapeutic drugs, including bortezomib.

The tubular injury observed in this case is a critical yet underexplored component of PGNMID pathology. Prior research has shown that tubular damage in monoclonal gammopathy-related kidney diseases often arises secondary to the toxic effects of excessive monoclonal light chain deposition within proximal tubular epithelial cells. This leads to complications such as lysosomal dysfunction, oxidative stress, and eventual cell apoptosis (1). These pathological changes are not only indicative of disease severity but may also serve as early markers of progressive renal dysfunction.

Emerging research supports the use of tubular injury markers, such as KIM-1 and NGAL, for providing prognostic insights and guiding therapeutic decisions, particularly in cases with significant tubular involvement (3). Despite this, the presence of tubular damage in PGNMID does not necessitate a change in the treatment approach or contraindicate the use of immunosuppressive agents like cyclophosphamide. Studies confirm that cyclophosphamide can be used safely in cases with tubular involvement, provided that renal function is meticulously monitored through regular assessments of serum creatinine, eGFR, and proteinuria levels (1).

The detection of tubular damage emphasizes the need for careful monitoring and potentially warrants the inclusion of adjunctive therapies aimed at reducing tubular stress and promoting repair. Nonetheless, treatment decisions should primarily be based on the overall severity and progression of the disease, rather than solely on the presence of tubular involvement.

Animal models, such as those employing Bence Jones proteins, are instrumental in elucidating the pathophysiological MGRS (17). In their study, Sirac et al. demonstrated the potential for rapid reversal of proximal tubular lesions in transgenic mice through Cre-recombinase-mediated deletion of pathogenic light chains from the mouse genome. This approach effectively replicated light chain-associated tubular toxicity, with significant resolution of tubular lesions observed within 2 months post-deletion.

Additionally, research by Lai et al. (18) involving the XBP1s transgenic mouse model highlighted the therapeutic potential of targeting heat shock proteins gp96/grp94. This intervention notably reduced the number of plasma cells in bone marrow and decreased the deposition of monoclonal IgG and kappa light chains in the kidneys, effectively mimicking the effects of proliferative glomerulonephritis linked to monoclonal immunoglobulin deposition.

These findings underscore the efficacy of targeting plasma cells to mitigate the production of nephrotoxic antibodies through chemotherapy, offering a promising therapeutic approach for conditions such as MGRS.

Initial treatment with rituximab, which targets B cells, was ineffective in improving serum creatinine or urinary protein levels because CD20 is not expressed on plasma cells. The renal biopsy findings, revealing monoclonal IgG3-kappa deposits in the glomeruli and kappa light chain deposits in the proximal tubules, suggested a pathology driven by plasma cells, despite the absence of detectable monoclonal proteins in serum and urine.

For patients with IgM deposits, the therapeutic strategy often targets the B-cell clone responsible for IgM production. Rituximab, a monoclonal antibody against CD20, is commonly employed because it effectively depletes CD20-positive B cells, thereby reducing IgM production. This approach leverages the understanding that IgM is predominantly produced by B cells and lymphoplasmacytic cells that express CD20 (19).

Although daratumumab, which targets CD38 on plasma cells, was initially considered due to its high efficacy in similar cases, it was not pursued because of its high cost (20). Instead, the VCD regimen was selected, targeting the plasma cell-driven pathology and demonstrating established efficacy in the treatment of MGRS.

Additionally, plasmapheresis has proven beneficial, particularly in scenarios involving acute kidney injury, as it can rapidly decrease circulating monoclonal proteins, providing immediate relief from symptoms (10). The substantial improvement observed after targeted therapy for plasma cell clones underscores the critical role of plasma cells in these conditions. Targeting plasma cell clones appears to be an effective strategy to reduce abnormal immunoglobulin deposition and control disease progression. These findings highlight the importance of personalized medicine in managing MGRS-related diseases.

## Conclusion

PGNMID and LCPT are distinct and complex subtypes of MGRS, it is especially rare when these two diseases occur simultaneously. Renal biopsy plays a critical role in the diagnosis of PGNMID and LCPT, informing tailored treatment approaches. The significant improvement observed in our case following the VCD regimen post-biopsy underscores the importance of targeted therapeutic strategies. Given the rarity and complexity of these conditions, ongoing research is essential. It will enhance our understanding and refine treatment methods, ensuring that patients benefit from optimized, personalized care.

## Data Availability

The datasets presented in this article are not readily available because of ethical and privacy restrictions. Requests to access the datasets should be directed to the corresponding author.
